# From Data to Optimal Decision Making: A Data-Driven, Probabilistic Machine Learning Approach to Decision Support for Patients With Sepsis

**DOI:** 10.2196/medinform.3445

**Published:** 2015-02-24

**Authors:** Athanasios Tsoukalas, Timothy Albertson, Ilias Tagkopoulos

**Affiliations:** ^1^Department of Computer Science and Genome CenterUniversity of California, DavisDavis, CAUnited States; ^2^Department of Internal MedicineUniversity of California, DavisDavis, CAUnited States; ^3^Northern California Veterans Administration Health Care SystemMather, CAUnited States

**Keywords:** sepsis, clinical decision support tool, probabilistic modeling, Partially Observable Markov Decision Processes, POMDP, CDSS

## Abstract

**Background:**

A tantalizing question in medical informatics is how to construct knowledge from heterogeneous datasets, and as an extension, inform clinical decisions. The emergence of large-scale data integration in electronic health records (EHR) presents tremendous opportunities. However, our ability to efficiently extract informed decision support is limited due to the complexity of the clinical states and decision process, missing data and lack of analytical tools to advice based on statistical relationships.

**Objective:**

Development and assessment of a data-driven method that infers the probability distribution of the current state of patients with sepsis, likely trajectories, optimal actions related to antibiotic administration, prediction of mortality and length-of-stay.

**Methods:**

We present a data-driven, probabilistic framework for clinical decision support in sepsis-related cases. We first define states, actions, observations and rewards based on clinical practice, expert knowledge and data representations in an EHR dataset of 1492 patients. We then use Partially Observable Markov Decision Process (POMDP) model to derive the optimal policy based on individual patient trajectories and we evaluate the performance of the model-derived policies in a separate test set. Policy decisions were focused on the type of antibiotic combinations to administer. Multi-class and discriminative classifiers were used to predict mortality and length of stay.

**Results:**

Data-derived antibiotic administration policies led to a favorable patient outcome in 49% of the cases, versus 37% when the alternative policies were followed (*P*=1.3e-13). Sensitivity analysis on the model parameters and missing data argue for a highly robust decision support tool that withstands parameter variation and data uncertainty. When the optimal policy was followed, 387 patients (25.9%) have 90% of their transitions to better states and 503 patients (33.7%) patients had 90% of their transitions to worse states (*P*=4.0e-06), while in the non-policy cases, these numbers are 192 (12.9%) and 764 (51.2%) patients (*P*=4.6e-117), respectively. Furthermore, the percentage of transitions within a trajectory that lead to a better or better/same state are significantly higher by following the policy than for non-policy cases (605 vs 344 patients, *P*=8.6e-25). Mortality was predicted with an AUC of 0.7 and 0.82 accuracy in the general case and similar performance was obtained for the inference of the length-of-stay (AUC of 0.69 to 0.73 with accuracies from 0.69 to 0.82).

**Conclusions:**

A data-driven model was able to suggest favorable actions, predict mortality and length of stay with high accuracy. This work provides a solid basis for a scalable probabilistic clinical decision support framework for sepsis treatment that can be expanded to other clinically relevant states and actions, as well as a data-driven model that can be adopted in other clinical areas with sufficient training data.

## Introduction

Over the past few decades, our society has transitioned to a state where bottlenecks have shifted from a lack of data, to limitations in extracting meaningful knowledge and subsequently use that knowledge to drive decisions. This data-rich, knowledge-poor oxymoron is particularly true in computationally-driven Clinical Decision Support Systems (CDSS), where advances in automated high-throughput data acquisition and electronic health records have yet to be translated into knowledge extraction and probabilistic decision guidance. This is true even in the cases of dangerous and ubiquitous threats to human health, one of which is sepsis. Sepsis is an overwhelming systemic immune response to infection, which results in damage to the patients’ own tissues and organs. This process can happen at any age, regardless of the underlying health condition and from seemingly benign incidents. Severe sepsis strikes about 18 million people annually (750,000 cases in the United States) and has a very high short-term mortality risk (28% to 50%) [1] Severe sepsis is the leading cause of Intensive Care Unit (ICU) deaths (60-80% of ICU deaths in developing countries) and it kills more than 6 million children world-wide every year [2].

Surprisingly, while sepsis is one of the most common diseases (more deaths than prostate cancer, breast cancer, and HIV/AIDS combined [3]), it has the lowest state-funding rates for research. This is in contrast to its severity, occurrence in our society (sepsis hospitalizations have more than doubled over the last 10 years [2]) and money spent to battle it (US $14.6 billion in 2008, an increase by an average of 11.9% each year). The diagnosis of sepsis is often delayed because it is difficult to differentiate from other high-risk conditions and this delay can lead to the rapid deterioration of the patient. One potentially transformative approach to this problem would be to exploit the vast amount of information that is hidden in the Electronic Health Records (EHR) of patients to derive a CDSS.

Adoption of EHR’s by health care systems was predicted to vastly improve the efficiency and quality of patient care [4]. Unfortunately, despite explosive EHR adoption, and enormous associated capital expenditures, these gains have yet to be realized [5,6]. One reason for this failure is that our capacity to utilize complex, large-scale data to generate knowledge and inform clinical decisions remains limited. For example, while CDSS have existed for decades, they are mostly limited to alert systems and (data-oblivious) agent-based suggestions that rely on hard-coded criteria. Although in certain systems patient cases are used for probabilistic training, these efforts focus on feature correlations and final clinical outcomes [7-10] rather than actionable policy (see [11,12] for a review). Our previous work on the associations among EHR observations for lactic acid prediction work falls also in this category (11).

Some of the most powerful methods for modeling decision making in clinical decision support are those that treat the learning problem as a Markov Decision Process (MDPs) [13]. A MDP is a discrete-time stochastic control process, where the next state depends only on the current state and the action that the decision maker performs, while it is conditionally independent of all other states and actions. An extension to MDPs are the Partially Observable MDPs, (POMDPs), where the states themselves are hidden and only observations are available. In that case, a belief regarding the current system state is formed based on the observations and their state-based likelihoods [14]. There are many methods to solve MDPs/POMDPs, including dynamic programming, linear programming and reinforcement learning [13-17]. When the problem becomes intractable, reinforcing learning methods are preferred as they do not require knowledge of the underlying MDP model.

Surprisingly, although the use of MDP methods in clinical settings is well established, there are only a few notable examples where POMDP has been explored for disease-specific decision support with probabilistic outcomes. MDP has been used for decision support in determining liver acceptance for liver transplants [18-20], HIV therapy initiation [21], breast cancer screening [22], treatment of hepatitis C [23], statin therapy timing [24], among others [25]. However, in most cases the pathology is complex, the underlying state of the patient is latent and we can only observe emitted signals (observations) with some uncertainty. A notable example of POMDP adoption in medical decision support is that of Hauskrecht and Fraser [26,27] who modeled Ischemic Heart Disease with a POMDP model that included both treatment and test actions. This work used an impressive hierarchical model for state variables and performed reasonably well in predicting optimal policies in that given scenario. Similarly, Turgay et al [28] used a POMDP model to support personalized mammography screening decisions in a model that used six states, three of which were fully observable. Kreke et al created a 2-state POMDP model for pneumonia-related sepsis patients that included only a Cytokine test as an investigative action and incorporated the Sequential Organ Failure Assessment (SOFA) score for the MDP state space definition [29,30]. In all these cases, both the state model and policy were course-grained, the parameter space limited and the training sets were a few dozen patients.

Here, we use a point-based POMDP solver together with a dataset of 1492 patients that is complete with time-stamped blood tests, vitals, and other relevant records for sepsis. We model patient trajectories and treatment by defining data-driven states, observations, probabilistic beliefs, actions, and rewards. We then evaluate the potential of this method to inform on optimal administration of antibiotic combinations, defined as antibiotic “policy”. In addition, we assess the predictive ability of applying machine learning methods to predict patient mortality and length of stay, in order to drive clinical decision support.

## Methods

### Data Collection

**Figure 1:**
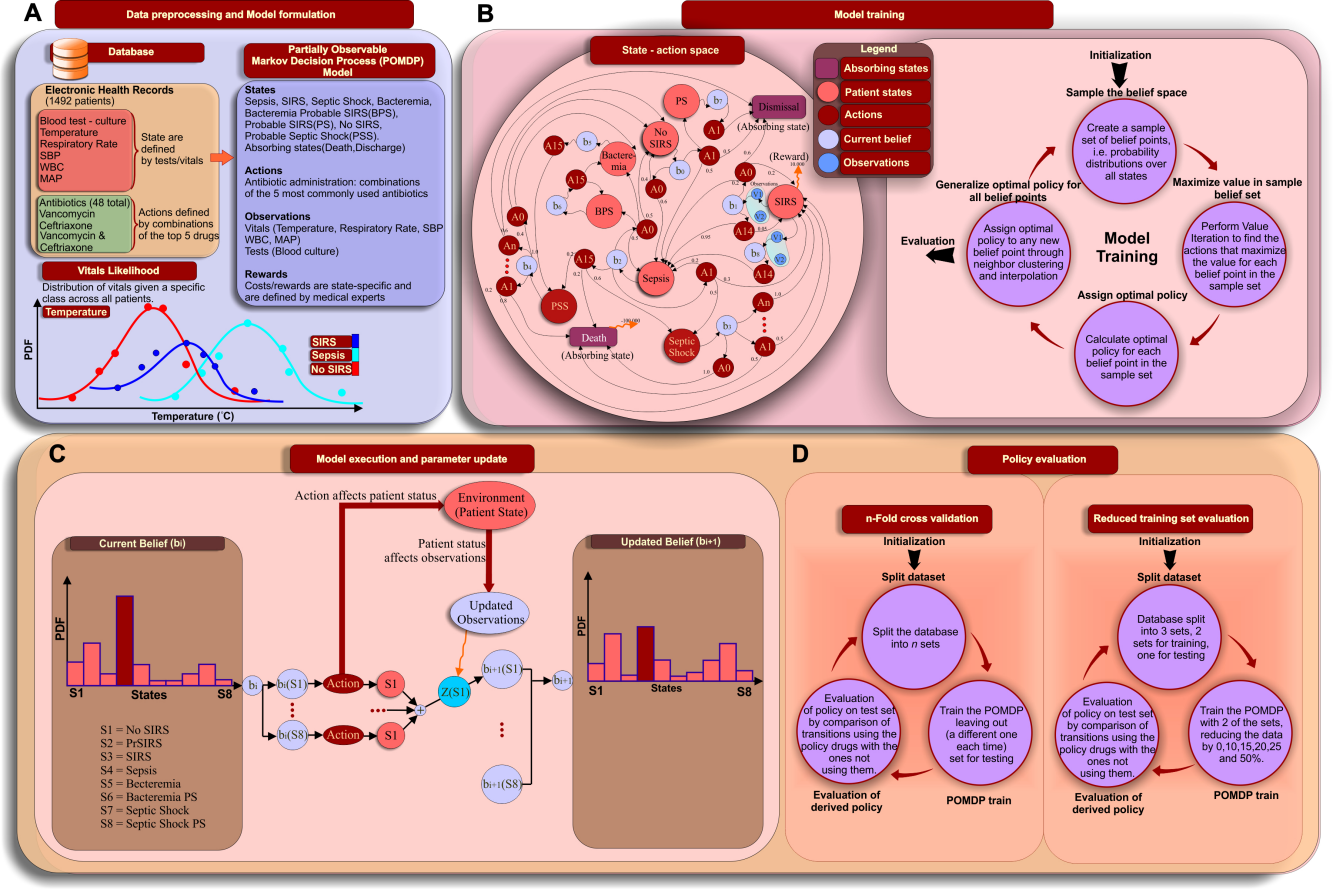
Development and evaluation of a clinical decision support system (CDSS) for Sepsis. (A) Synopsis of the EHR database, distribution of vitals, states, actions. Likelihood functions were used to calculate state-specific transition and observation probabilities. (B) The state-action diagram describes underlying patient states, possible transitions and beliefs based on the values of the observed variables. There are two absorbing states, “Death” and “Dismissal”. The training of the POMDP model that is applied on the state-action space performs value iteration updates on a sample set of beliefs, effectively using a Monte Carlo approach for sampling together with dynamic programming for the calculation of the value iteration. (C) Belief (ie, probability distribution of the patient states) is updated based on the action taken and the new observations. At each time step patient vitals are observed and the action that corresponds to the optimal policy is taken. A new set of observations (vitals, tests) will lead to an updated belief that may lead to a new action to be undertaken. The update is asynchronous, as it is calculated on-the-fly as new information arrives. (D) Evaluation of the CDSS framework was performed through 5-fold cross validation and data size sensitivity analysis.

**Figure 2:**
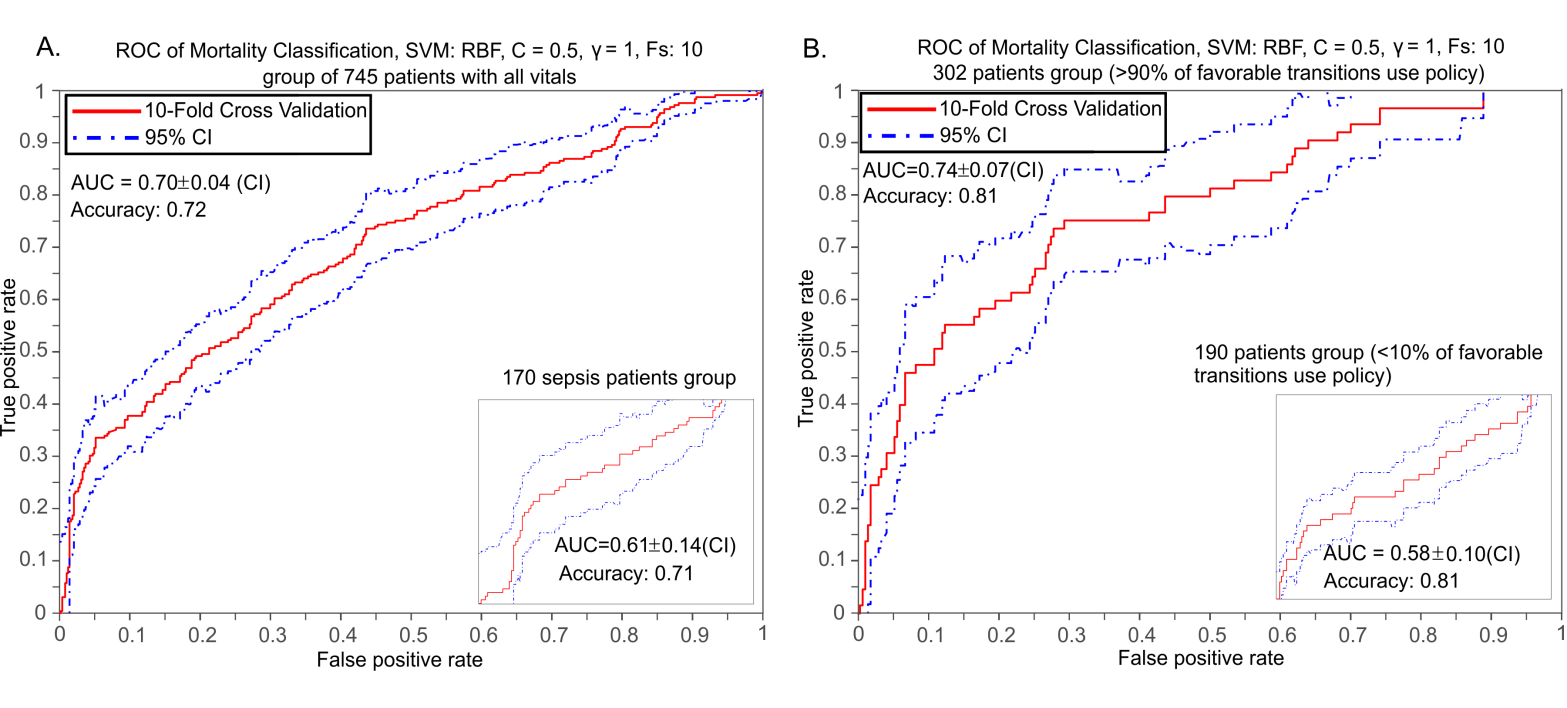
ROC curves of mortality classification/prediction. Support Vector Machine training results, given the provided vitals and mortality model, in a 10-fold cross validation scheme. The features used for the classification are temperature, respiratory rate, WBC, MAP and lactate levels and 745 patients for whom all seven variables were available were considered out of the 1492 of the DB. Radial Basis Function (RBF) kernel was used for the SVM training. The five measurement variables were summarized by their mean and standard deviation (STD) across the trajectory of each patient. Principal Component Analysis (PCA) was also used to assess whether linear transformation of the feature space and dimensionality reduction can be achieved in this case. A filter method was applied using the Area Under the Curve (AUC) of the Receiver Operating Characteristic (ROC) curves as a ranking criterion. The positive/negative classes for mortality prediction are defined as alive/deceased respectively. (A) Classification using the patients that have all features available. The maximum prediction accuracy when all patients with available all vitals are used, is 0.72 (72%) and the AUC is 0.70. (Inset) Classification using the 170 patients that have a diagnosis related to Sepsis. The maximum prediction accuracy is 0.71 (71%) and the AUC is 0.61. (B) SVM classification performance when the patient group that have ratio of transitions to better states with policy drugs vs all transitions to better states > 0.9 (302 patients out of 745) is used. (Inset) SVM classification performance for the patient group that have ratio of transition to better states with policy drugs vs all transitions to better states less or equal to 0.1 (190 patients out of 745).

An EHR database containing 1492 adult patients (≥18 years of age; ICU cases) with personal health information removed and meeting at least two Systemic Inflammatory Response Syndrome (SIRS) criteria [31] admitted to the University of California Davis Health System (UCDHS), was used for all the analyses, Figure 1 (a). Informed consent was obtained for all human subjects and the analysis was approved by the institutional review board of the University of California, Davis (IRB # 254575). Of the 1492 patients, 45.0% were female, the mean length of stay was 17.0 (SD 36.7) days, and 38.0% were admitted from the emergency department. Table 1 summarizes the dataset used; Figure 2 provides a histogram of the total/ICU length of stay). UCDHS is a tertiary care, academic medical center that did not have an active EHR alert system for the diagnosis or treatment of sepsis during the study period. All data were abstracted retrospectively from the EHR via structured query language interrogation of a de-identified relational database. Patients were included in the database if they were hospitalized and discharged between 1 January 2010 and 31 December 2010. The following six variables were used as *observation variables* in our model Figure 1 (a): temperature, respiratory rate (RR), white blood count (WBC), mean arterial pressure (MAP), systolic blood pressure (SBP), and blood culture results, with explicit mention of the bacterial species that were present in the culture. The first five variables are measurements of a patient’s condition recorded over time; temperature, RR, WBC, and MAP are part of the SIRS criteria.

**Table 1:** Database characteristics.

States and state transition distribution in the dataset		
State	Transitions (4200 total)	Patients (1492 total)
No SIRS	1300 (30.85%)	915 (61.33%)
SIRS	294 (6.98%)	264 (17.70%)
Sepsis	41 (0.97%)	38 (2.54%)
Septic Shock	17 (0.40%)	17 (1.14%)
PS (Probable SIRS)	1929 (45.79%)	939 (62.94%)
Bacteremia	157 (3.73%)	121 (8.11%)
BPS (Bacteremia Probable Sepsis)	323 (7.67%)	179 (11.99%)
PSS (Probable Septic Shock)	139 (3.30%)	135 (9.05%)
Demographics and Final Diagnosis		
Characteristic	Value	
		
Female Gender	677 (45.37%)	
Male Gender	815 (54.63%)	
Mortality Rate	376 (25.2%)	
Length of Stay (days)	Mean: 17 Median: 8	
Intensive Care Unit Stay (days)	Mean: 6.5 Median: 1	
Sepsis Diagnosis	188 (12.60%)	
Septic shock Diagnosis	21 (1.41%)	
Severe Sepsis Diagnosis	8 (0.53%)	

### Sepsis Model

### State Definition

The states that we include in our formulation were selected based on the existence of well-defined criteria and expert opinion, Figure 1 (a). Each of these states is defined by a number of features as illustrated in Table 2. The SIRS criteria that define the respective state are HR >90 beats per minute, RR >20 breaths per minute (or partial pressure of arterial CO_2_ < 32mm Hg), temperature either >38°C or <36°C, and WBC either >12,000 or <4000 cells/mm3 (or > 10% bands). For a patient to be diagnosed with sepsis, at least two of the SIRS criteria need to be present and a suspected infection should be present (eg, evident through blood test results). Septic shock occurs when there is sepsis-induced hypotension (where either SBP is below 90 mm Hg, < 40 mm Hg below baseline, or MAP is below 70 mm Hg) that persists despite adequate fluid resuscitation. Additionally, we included states that cannot be fully determined during the training phase because of missing database information, such as missing vitals measurements and time stamps. These states are “Probable Septic Shock” (PSS: Hypotention, positive blood test and no adequate vitals to determine SIRS and/or Sepsis), “Probable SIRS” (PS: no infection and no vitals to determine SIRS), “Bacteremia Probable SIRS” (BPS: infection but no vitals to determine SIRS and/or Sepsis). We have not included the “Severe Sepsis” state in this work, as the current dataset does not offer enough information required to incorporate the organ failure into the patient state definition.

**Table 2:** States and their definition based on vitals and blood tests.

State	Features
No SIRS	
SIRS	heart rate (HR) >90 beats per minute
	respiratory rate (RR) >20 breaths per minute (or partial pressure of arterial CO2 < 32)
	temperature either >38°C or <36°C
	white blood cell count (WBC) either >12,000 or <4000 cells/mm3 (or > 10% bands)
Sepsis	SIRS and Infection (blood test result)
Severe Sepsis	Sepsis and Organ failure (shown in ICL code)
Septic Shock	Sepsis and Hypotension (systolic blood pressure (SBP) is below 90 mm Hg, < 40 mm Hg below baseline, or the mean arterial pressure (MAP) is below 70 mm Hg)
	
PS	No infection– no vitals to determine SIRS
Bacteremia	Infection (blood test result) & No SIRS
BPS	Infection – no vitals to determine SIRS (thus Sepsis)
PSS	Hypotension, positive blood test and no vitals to determine SIRS (thus Sepsis)

### Actions

A policy is one or more actions that are followed. Each antibiotic combination is considered a possible action. A total of 48 antibiotics have been included in the patients’ EHR that we analyzed. Here we consider the top five more frequently used antibiotics (Vancomycin, Cefepime, Metronidazole, Ceftriaxone, and Meropenem) and all their possible combinations, plus one more combination that encapsulates all other antibiotics that may have been used. This results in 32 possible combinations, each one defining a different action of the five most frequently used antibiotics. Vancomycin is a glycopeptide antibiotic that inhibits the cell wall synthesis of gram-positive bacteria, although it is avoided due to its nephrotoxicity and ototoxicity. Both Cefepime and Ceftiaxone are cephalosporin antibiotics that have activity against both Gram-negative and Gram-positive bacteria and they are especially used to treat moderate-severe pneumonia. Cefepime is also used to treat infections caused by multi-drug resistant microbial strains. Metronidazole is a nitroimidazole antibiotic that is used particularly for anaerobic bacteria and some protozoa. Meropenem is an ultra-broad spectrum antibiotic and beta-lactam that inhibits bacterial wall synthesis. Combinations of all states with all possible actions comprise the state-action space for our sepsis model, Figure 1 (b, left side).

### Rewards

**Figure 3:**
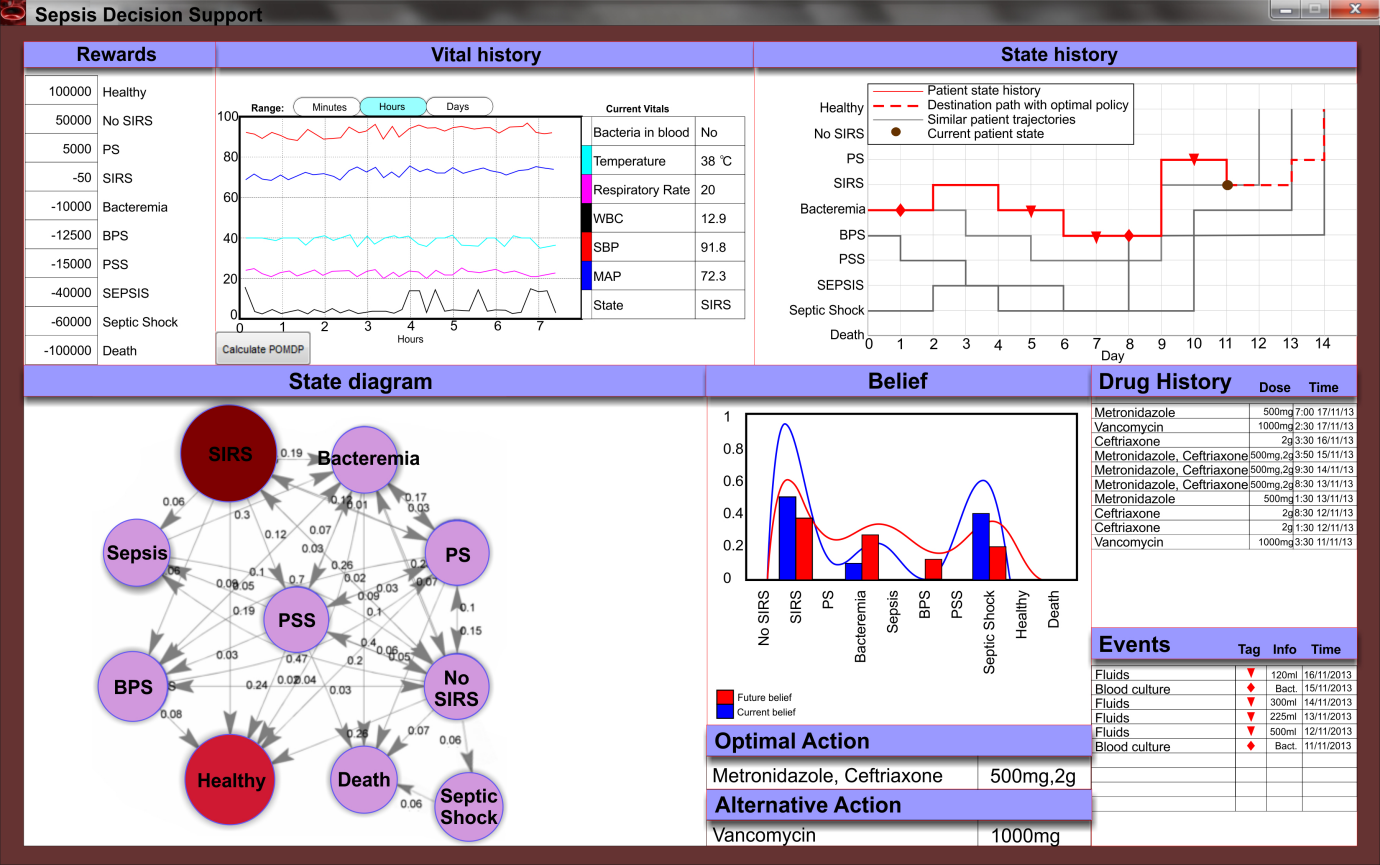
A graphical user interface (GUI) for the clinical decision support system. Physicians have access to real-time and historical vital history (upper left), as well as state history (upper right) for a given patient. The state history displays events, most likely path if optimal policy is to be adopted and the past trajectories of the top three patients that had similar profiles (ie, past states and vitals) to the current patient. The state diagram (bottom left) depicts the state transition probabilities and its updates based on the current state. The GUI also displays the belief distribution, optimal action given the current belief and the second best alternative action. Drug history and significant events are displayed (bottom right). The tool updates values automatically with new data, although the user has the flexibility to revise the desired reward values (upper left) and then manually trigger a recalculation of the optimal policy.

Reward/cost values have been provided empirically by physicians, based on the severity of each state. These are, from best to worse: Healthy (100,000), No SIRS (50,000), Probable Sepsis (PS, 5000), SIRS (-50), Bacteremia (-10,000), Bacteremia Possible Sepsis (BPS, -12,500), Possible Septic Shock (PSS, -15,000), Sepsis (-40,000), Septic Shock (-60,000), Death (-100,000). This information is also depicted in Figure 3, first panel.

### Calculation of Transition and Observation Probabilities

Transition probabilities are calculated as the frequency of State-Action-Next State patterns in each State-Action combination. Similarly, observation probabilities are calculated based on the frequency of observing vital value (in bins) combinations in any given state. In order to extract the probability of the observation combinations, we fit the distribution that best describes the data for each vital and state and then divide it in five non-overlapping, equally-sized bins. The number of bin was selected such that sufficient data (>10 samples) will be present in each bin. For each state, the distributions of the five vitals are divided in five equal parts across their min-max range (each assigned a probability from the distribution) with blood test being modeled with a binary variable (presence/absence of bacteria). This leads to 6250 combinations (2∙5^5^).

### POMDP Formulation

**Figure 4:**
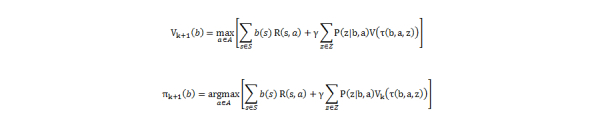
Value iteration calculates the value of each state by solving the Bellman equations.

A POMDP is defined as a 8-tuple (*S, A, Z, T, O, R, b_0_, γ*), where *S* is a finite set of states, *A* is a finite set of actions, *T:S × A × S → P(S)*is the state transition function, *T (s, a, s’)* denotes the probability *P(s’| s, a)* of reaching state s’ from state by taking action *a*, *R: S × A → ℜ* is the reward function, *P(s’s, a)* denotes the immediate reward of executing action *a* in state *s, γ ∈ [0, 1]* is the discount factor, *Z* is the finite set of observations, *O:S × A → P(S)* is the observation function, *O(s, a, z)* denotes the probability *P(z|s, a)* of perceiving observation *z* when taking action *a* and arriving in state *s*, *b_0_* is the initial state probability distribution, *b* is the state probability distribution and *b_0_(s)* denotes the probability of starting in state *s*. A policy *π* for a POMDP problem is defined as *π(b)→a* and its value is the expected cumulative discounted reward that we will receive if we perform actions *a* when we have belief *b*. The policy that maximizes this cumulative value is called the *optimal policy*. We used a POMDP model training methodology that is based on a Monte Carlo approximation for solving the Value Iteration method over a sampling belief space, Figure 1 (b, right side). Value iteration calculates the value of each state by solving the Bellman equations (Figure 4).

**Figure 5:**
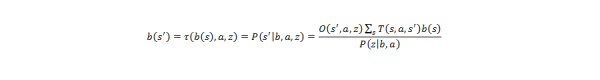
Belief b(s) of being in state.

If we have belief b(s) of being in state s, perform action *a* and we observe *z*, the updated belief for being in state *b(s’)* is shown in Figure 1 (c) and Figure 5.

### POMDP Statistical Evaluation

We use *Perseus* [32], a randomized point-based value iteration algorithm to extract the optimal policy with default settings. We use 5-fold cross-validation to evaluate the generalization error of the policy, Figure 1 (d). For a specific transition, a CDSS-derived policy is deemed as followed if one or more of the antibiotics in the policy have been administered to the patient. Comparison is performed between treatments that are in agreement with the CDSS-derived policy for each transition and those that are not (non CDSS-derived policies), measuring the percentage of transitions that lead to a better/same/worse state in both cases. At a trajectory level, we compare what percentage of trajectories move to a better state on average with and without the CDSS-derived policy. The robustness of the algorithm to perform in the context of reduced data is tested by splitting the patient data into 3 parts, where only the 2 out of the 3 parts are used for training. The algorithm is trained on randomly selected subsets of the 2/3 training data that correspond to the desired percentage of the total training set. The performance is always evaluated in the same testing set (ie 1/3 of the total dataset) and the whole process is repeated 10 times to reduce bias, Figure 1 (d). All *P* values are calculated using hypergeometric distribution with multiple hypothesis correction (Benjamini-Hochberg).

### Length-of-Stay and Mortality Inference

For the length of stay (LOS) prediction, we first split the patients in equally-sized bins, based on their LOS distribution, thus maximizing entropy and to avoid bias on the training data. Then we used Support Vector Machines (SVM) with different kernel functions [33,34]. In SVM classification, the training feature vectors are mapped to a higher dimension space, in which the SVM determines a linearly separating hyperplane given by a maximal margin [35]. In order to predict survivability results given a patient’s vital signs, an SVM classification method was used, taking into account five features (temperature, respiratory rate, WBC, MAP, and lactate levels) and the mortality state of each patient, for both binary and multi-class classification. For the latter, we consider both combinatorial pairwise and one-versus-all schemes [36], where we observed no significant difference in our results. We evaluated the performance of the classifier by performing cross-validation (CV) and calculating the Receiver-Operator-characteristic (ROC) curves, confidence intervals (CI), and the area-under-the-curve (AUC).

## Results

### Optimal Policy Prediction by Data-driven Machine Learning Approaches

**Figure 6:**
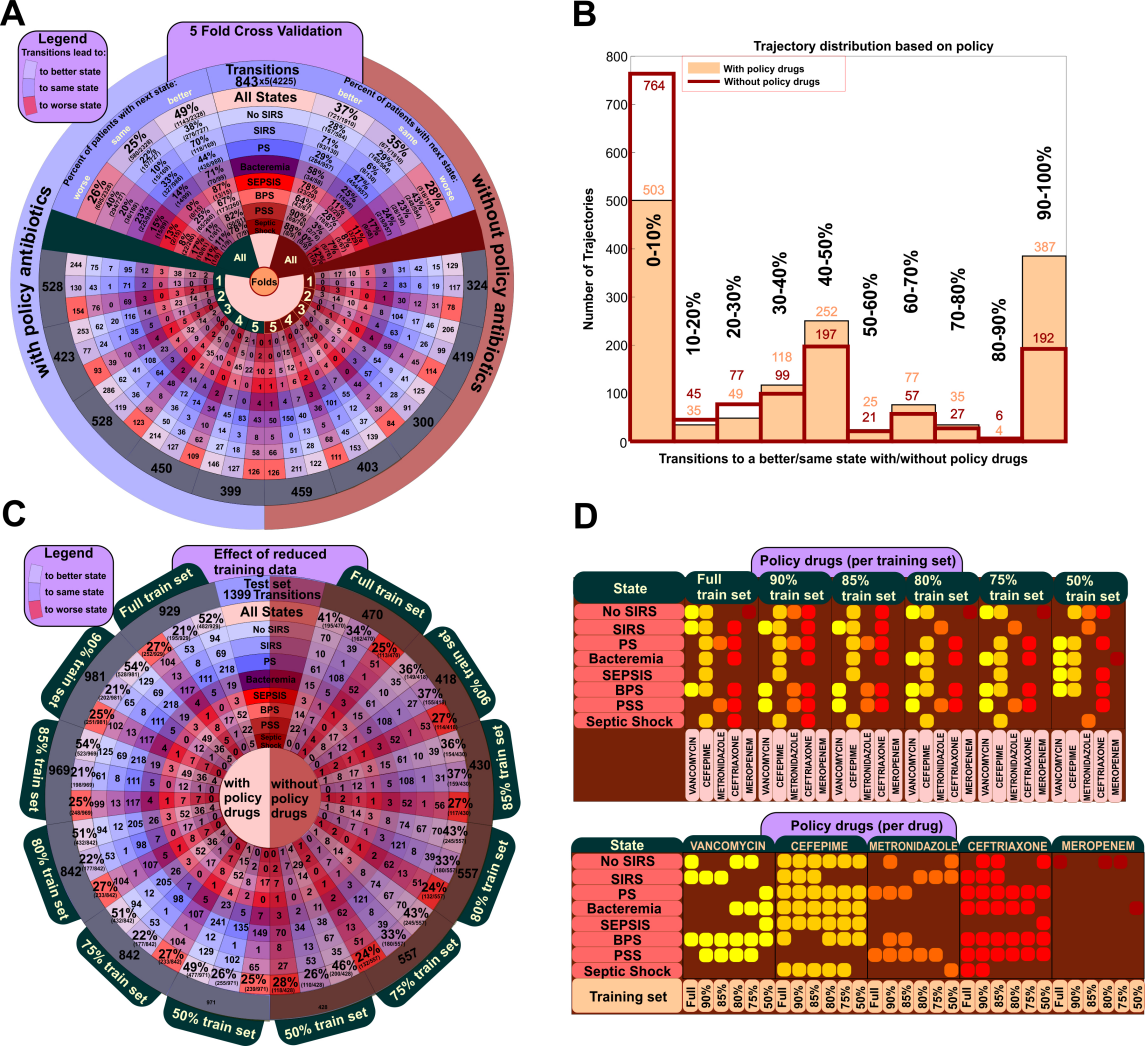
Performance and Robustness of the POMDP Clinical Decision Support System for Sepsis. (A) 5-fold cross-validation results depict the performance of each fold with (left, blue) and without (right, brown) using the policy-proposed antibiotic combination. Each cell contains the number of transitions (4225 total transitions; 843 transitions per test fold) that lead to worse, equal or better states in each case. A state-specific percentage across all folds allows for comparison between the different policy strategies. (B) Number of patient trajectories vs. the percentage of their transitions that lead to a better state. (C) Dependency of CDSS performance on data size based on stratified reduction of the dataset. Outcome is shown for policy-proposed antibiotic combinations (left) and all other combinations (right) for different states (D) Changes in antibiotic combinations proposed in calculated optimal policy as a function of data-size reduction. Each row is a state and each column represents a drug-training set combination. The two tables depict which drug combinations were found to lead to better outcomes when in the perspective state, in the general case. States are as defined in the Methods sections, with three states denoting uncertainty due to missing data (PS: probable sepsis; BPS: bacteremia, probable sepsis; PSS: probable septic shock).

We performed 5-fold cross validation (CV) to evaluate the generalization error of our approach, with similar results across all folds, Figure 6 (a) and Tables 2 and 3. The CDSS-derived optimal policies in each transition led in significantly more occurrences to better states than when the treatment that was followed by the physicians was not in agreement with the CDSS-derived policy (49% of transitions to better states when the CDSS-derived policy was followed vs 37% otherwise; *P*=1.3e-13). Interestingly, when non-CDSS policies were used, patients tend to stay in the same condition (35% in non-CDSS policies vs 25% in CDSS policies, *P*=5.1e-13) while the difference between CDSS-derived policies and non-CDSS policies is not statistically significant for transitions to a worse condition (28% in non-CDSS policies vs 26% in CDSS policies, *P*=4.2e-1). We then analyzed each patient trajectory independently, to estimate the number of transitions within a trajectory that lead to better states, with and without following the policy. Results show that when the optimal policy prediction (ie, the policy that maximizes the expected cumulated reward, as defined in the methods section) is followed, there exists a significant shift to trajectories that have more than 90% of their transitions leading to a better state, Figure 6 (b). When the policy was followed, 387 patients (25.9%) have 90% of their transitions to better states and 503 patients (33.7%) patients had 90% of their transitions to worse states (*P*=4.0e-06), while in the non-policy cases, these numbers are 192 (12.9%) and 764 (51.2%) patients (*P*=4.6e-117), respectively. Furthermore, the percentage of transitions within a trajectory that lead to a better or better/same state are significantly higher by following the policy than for non-policy cases. Indeed, 605 versus 344 patients (*P*=8.6e-25) have 90% of their transitions to a better state with versus without following a CDSS-derived policy, Figure 1 (a). This result was observed in all five runs of the 5-fold CV and on the full dataset, hence it holds for different data distributions.

**Figure 7:**
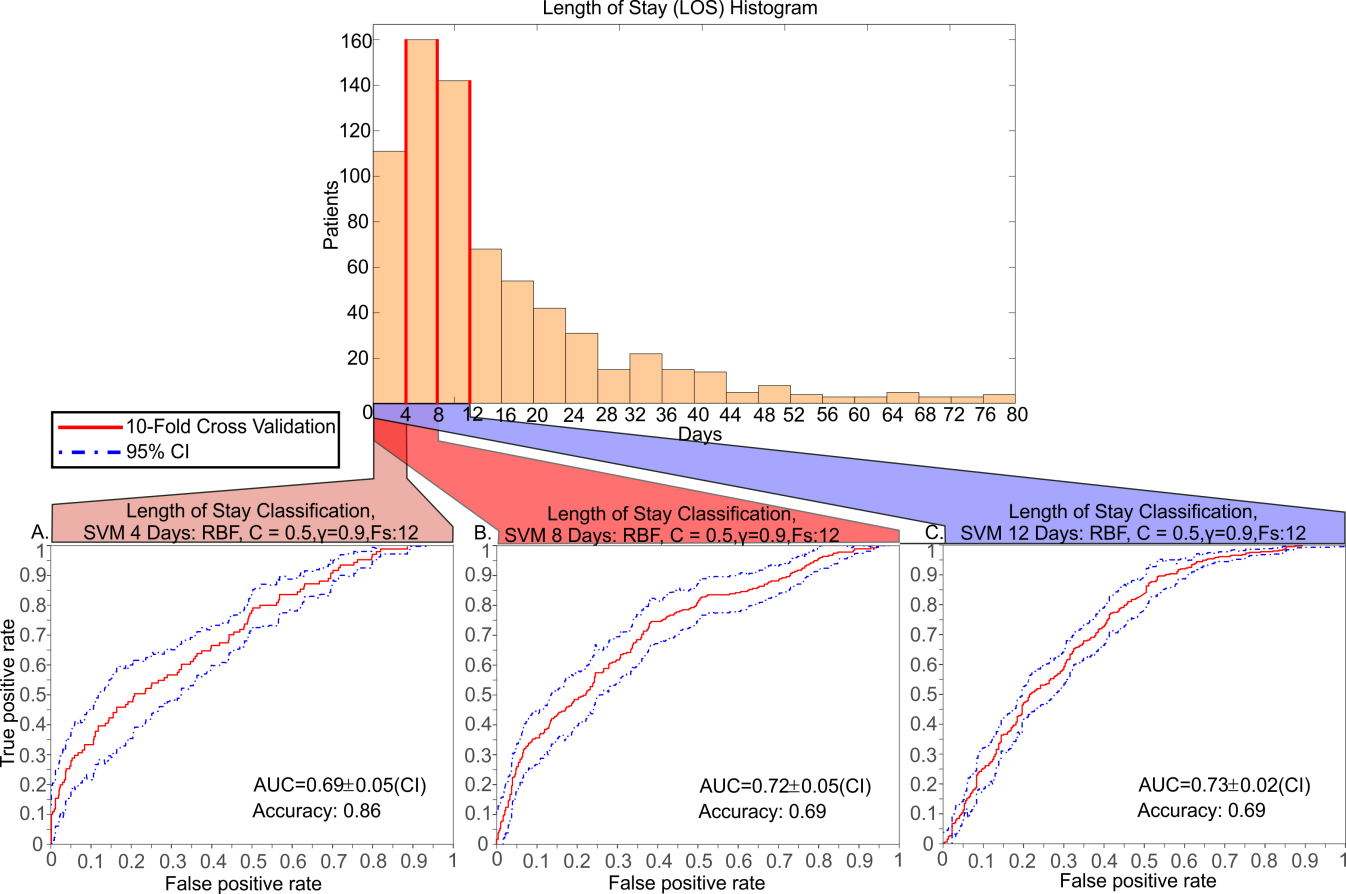
Predicting a patient’s Length of Stay (LOS). Histogram of the LOS for the 745 patients in the database that have a complete record (median length of stay is 10.4 days). ROC curves for binary classifiers when 4, 8 or 12 days was used as the boundary between the two classes.

Next, we evaluated the robustness of the POMDP framework to decreasing sets of training data. To perform this analysis, we iteratively reduced the training set and we evaluated in the same testing data set (see Methods). Results demonstrate the method is robust to decreasing amount of data in the set, Figure 6 (c) as well as Figures 3 and 7, mainly due to the significant overlap of the various antibiotics in each combination proposed by the optimal policy. To gain more insight on how the policy changes by decreasing the training set we constructed a comprehensive map of the optimal policy for each state, Figure 6 (d). The resulting map provides the CDSS-derived drug combination that led to more favorable outcomes in each state, Table 3. It is important to note, that these policies correspond to a definite knowledge that the patient is that specific state (belief/probability of 1), which is almost never the case as his/her prior history (previous states, clinical information, etc) shapes the belief distribution across all states at any given time. Additionally, the depicted drug combinations are associated with a better outcome overall, and not that are necessarily the optimal combination under any condition when a patient is in that specific state, since potent drug combinations are used to more severe cases, which have a higher probability to transition to a worse state. The optimal decision for any state will ultimately be a function of all observations (vitals, blood results, etc). Their associations will depend on the structure of the data that were used for the CDSS training.

**Table 3:** Optimal Policy based on from the POMDP CDSS tool. Note that this result assumes that current state is known and given in the state column (Belief/Probability of 1).

State	Drugs
SIRS	CEFEPIME,METRONIDAZOLE,CEFTRIAXONE
PS	METRONIDAZOLE,CEFTRIAXONE,MEROPENEM
Bacteremia	CEFEPIME,CEFTRIAXONE
Sepsis	CEFTRIAXONE
BPS	VANCOMYCIN,CEFEPIME,CEFTRIAXONE
PSS	METRONIDAZOLE,CEFTRIAXONE
Septic Shock	CEFTRIAXONE

### Mortality Prediction

Clinical outcome was found to be accurately classified by using Support Vector Machines (see Methods). In order to predict patient mortality, we used the five features (temperature, respiratory rate, WBC, MAP, and lactate levels) as well as the final outcome for each patient. This led to a dataset of 745 patients out of the 1492 total for whom all six variables were available. The five measurement variables were summarized by their mean and standard deviation (STD) across the trajectory of each patient [37]. Principal Component Analysis (PCA) was also used to assess whether linear transformation of the feature space and dimensionality reduction can be achieved in this case [38]. A filter method was applied using the Area-Under-the-Curve (AUC) of the Receiver Operating Characteristic (ROC) curves as a ranking criterion [39]. Radial Basis Function (RBF) kernel was used for the SVM training in a 10-fold cross validation scheme. The positive/negative classes for mortality prediction are defined as alive/deceased, respectively. Mortality classification of 745 patients has an AUC of 0.70 (SD 0.04; 95% CI) and accuracy of 0.72, Figure 2 (a). When the test set focuses on the 170 patients with sepsis group, it achieves an AUC of 0.61(SD 0.14) and accuracy of 0.71, Figure 2 (a). For the group of patients with a better-to-all transition ratio larger than 0.9 (302 patients out of 745) the AUC is higher at 0.74 (SD 0.07) and the accuracy is 0.81, Figure 2 (b), while AUC drops to 0.58 (SD 0.10) for patients with a ratio of better-to-all smaller than 0.1. Comparison of these results argue that the trained classifier performs better and is more accurate in cases where the proposed policy has been used and its discrimination power is higher when these policies lead to a favorable outcome.

### Length of Stay Prediction

To predict the length-of-stay (LOS), we trained SVM classifiers with two extra features that were found to be informative: the occurrences of a positive blood culture and the number of policy drug administration during a patient’s stay. We then defined and classified the patients in two classes, based on the length of stay. The threshold for that discrimination was driven by the median length of stay in the hospital (10.4 days) and hence we selected thresholds of 4, 8 and 12 days. A 10-fold cross validation scheme was used for the evaluation of the classifier. The AUC of the classifiers were 0.69 to 0.73 with small deviations in the CI (0.02-0.05) and accuracies from 0.69 to 0.82, Figure 7. Multi-class classification for multiple length-of-stay bins (0-3, 3-6, 6-12, 12+ days) had similar results, although the AUC drops to 0.53 when predicting the two intermediate (3-6 and 6-12) classes, Figure 6.

## Discussion

In this work, we used the EHR of 1492 patients to build a decision support tool and predictive classifiers for patients with sepsis. Despite the fact that the dataset was limited in both number of patients and features available, the CDSS methodology resulted in data-driven policies that led to significantly improved patient outcomes. Similarly, we demonstrated that time-stamped EHR observational data, such as patient vitals and blood results, can be used to predict mortality and length of stay intervals, with increased accuracy and discriminative performance.

Given the vast combinatorial space of treatments and outcomes, one of the main challenges in the development of a statistical decision support tool is the definition of states and actions in a way that is both clinically relevant and computationally feasible. To create a framework that balances these trade-offs, we used expert knowledge and statistical methods to efficiently represent clinical cases within the POMDP framework, while at the same time making sure that each state-action combination has sufficient data for model training and testing. As the size of clinical databases scales up, an automated state and action definition technique can be applied, which might lead to interesting insights on what is medically relevant in each case. Our robustness analysis argues that a POMDP-based tool is quite robust even for small sample sizes and it remains to be seen the generalization boundaries of such approach for larger, integrative datasets, more sophisticated state-action spaces with additional features, and complex clinical histories. As with any data-driven predictive approaches, the generalization error and applicability of the results is dependent on the extend a model can capture the real state and action space, as well as the various biases that arise due to limited sample sizes, data quality and precision. For instance, differences in patient populations, microbiological resistance patterns of the wards, anti-infective pre-treatment of patients, administration of drugs (eg, vasopressors) or treatments (e,g, ventilator support) that are currently not captured limit the applicability of this study as they can substantially change the proposed policies and actions. To address these issues, the methods that are proposed here can be applied in larger datasets that can support a more extensive modeling for states, actions and observables, while correcting for possible biases across different attributes.

This initial study paves the way for several interesting directions towards a predictive CDSS for sepsis treatment. In addition to the SIRS criteria and the indication of an infection, it would be useful to take into account possible dysfunctional organs, a set of information that was not present in the database that we used here. As such, we can define nine states for organ dysfunction: absent, Respiratory, Coagulation, Liver, CNS, Renal, Metabolic, Cardiovascular, Multi-organ dysfunction (the latter defined as two or more organ failures). The Sequential Organ Failure Assessment (SOFA) criteria and score [40] can be used for this purpose. The action space can similarly be expanded to include several other actions that are important for sepsis treatment, such as the administration and dosage of IV fluids, vasoactive medications, initiation of mechanical ventilation, oxygen therapy, hemodialysis, the use of sepsis order set, and other admission and/or transfer decisions. To this end, a more extensive dataset, both in terms of features and patients is crucial so that the state/action combinatorial space will have adequate training samples. Furthermore, it is important to extend the number of composite features that one will investigate for complex traits, as in this study we only considered up to seven features. Such extension would likely lead to more accurate predictions regarding mortality and patient’s length-of-stay. From a technical perspective, it would be important to work towards an algorithmic framework that can distinguish patients that have reached a state from different trajectories, as the optimal treatment in each of these scenarios can be substantially different. Although this would violate the Markov property in the general case, one can investigate finite-memory models that can accommodate such setting.

Finally, an important aspect of any CDSS tool is an intuitive and interactive visualization of the patient status, past history and decision space. For this work, we have developed an interactive graphical user interface (GUI) that is connected with the POMDP solver and the database and can display the vitals, drug and state history, state belief, state diagram with all possible transitions and their probabilities, as well as the optimal/near-optimal actions given the current belief for the patient’s state, Figure 3. The user can also define the rewards for each state and re-calculate the POMDP-derived optimal policy. In addition, the patient’s trajectory is compared on-the-fly with other patient’s trajectories in the database for comparison and visualization of potential outcomes. Novel visualization methods and interactive tools, such as head-mounted displays that are non-obtrusive are promising candidates to pair with the proposed CDSS, both as display and acquisition devices. The ultimate goal should be to use real time learning and analysis obtained from readily available EMR data, to warn the clinician of important changes in patient “states” and the need for new “actions” to improve the outcome of severe sepsis patients. As such, the integration of “big data” analytics with ubiquitous computing has the potential to revolutionize emergency and intensive care medicine as we know it.

## Multimedia Appendix 1

Supplementary Tables and Figures.

## Abbreviations

AUC: area under the curve
BPS: bacteremia probable SIRS
CDSS: clinical decision support system
CI: confidence interval
CV: cross-validation
EHR: electronic health records
ICU: intensive care unit
LOS: length of stay
MAP: mean arterial pressure
MDP: Markov Decision Process
PCA: principal component analysis
POMDP: Partially Observable Markov Decision Process
PS: probable SIRS
PSS: probable septic shock
RBF: radial basis function
ROC: receiver-operator-characteristic
RR: respiratory rate
SBP: systolic blood pressure
SIRS: Systemic Inflammatory Response Syndrome
SOFA: sequential organ failure assessment
SVM: support vector machines
UCDHS: University of California Davis Health System
WBC: white blood count
