# Supplementary material for: From Data to Optimal Decision Making: A Data-Driven, Probabilistic Machine Learning Approach to Decision Support for Patients With Sepsis
Source: JMIR Med Inform. 2015 Feb 24;3(1):e11. doi: 10.2196/medinform.3445 (PMC4376114; doi:10.2196/medinform.3445)
Supplement: Supplementary file 1 [file medinform_v3i1e11_app1.pdf]

From Data to Optimal Decision Making: A Data-driven, Probabilistic Machine Learning Approach to Decision Support for Patients with Sepsis

Athanasios Tsoukalas[1,2], Timothy Albertson[3,4], Ilias Tagkopoulos[1,2*]

[1] UC Davis Genome Center, University of California-Davis, Davis, CA, USA.
[2] Department of Computer Science, University of California-Davis, CA, USA.
[3] Department of Internal Medicine, University of California, Davis, CA, USA
[4] Northern California Veterans Administration Health Care System, Mather, CA, USA
*Corresponding author

Supplementary Online Material

Supplementary Table 1. Summary and p-values of 5-fold policy CV. Number of patient trajectory steps that lead to a better, same or worse state, with and without the optimal policy.

	with policy	without policy	**p-value**
Transitions to better state with policy drugs vs to better state without			
to better state	1143	721	
to other states	1185	1189	**1.28e-13**
Transitions to worse state with policy drugs vs to better state without			
to worse state	605	518	
to other states	1723	1392	**4.21e-1**
Transitions to same state with policy drugs vs to same state without			
to same state	580	671	
to other states	1748	1239	**5.14e-13**
Trajectories (with >90% of transitions leading to a better state with/without policy drugs)			
to better -same > 90%	387	192	
to better -same <90%	1098	1293	**1.34e-19**
Trajectories (with >90% of transitions leading to a better-same/worse state with policy drugs)			
90% of transitions to better-same	387	1098	
90% of transitions to worse	503	982	**4.01e-06**

Trajectories (with >90% of transitions leading to a better-**same** /worse state without policy drugs)

90% of transitions to better-same	1293	192	
90% of transitions to worse	721	764	**4.63e-117**
Trajectories (with >90% of transitions leading to a better/same state with/without policy drugs vs all to better/same leading states)			
above 90% of transitions to better-same	605	344	
below 90% of transitions to better-same	880	1141	**8.65e-25**

Supplementary Table 2. 5-fold cross-validation results for evaluating the performance of the method, given the actual patient transitions with and without using the policy-proposed antibiotic combination. The table contains the number of transitions (4225 total transitions; 843 transitions per test fold) that lead to worse, equal or better states for each case. This table corresponds to Fig. 2A.

	next state with policy antibiotics															next state without policy antibiotics														
	better					same					worse					better					same					worse				
**FOLD State**	1	2	3	4	5	1	2	3	4	5	1	2	3	4	5	1	2	3	4	5	1	2	3	4	5	1	2	3	4	5
No SIRS	75	62	62	50	27	43	23	36	36	18	76	39	59	62	58	15	46	41	37	28	19	26	28	45	51	31	45	38	68	66
SIRS	7	20	41	36	14	1	1	7	3	3	0	10	8	10	8	42	17	9	12	13	6	1	1	1	0	12	4	2	5	5
Probable SIRS	95	116	108	72	45	71	35	64	74	83	69	31	48	34	43	31	104	54	55	40	83	63	57	101	150	30	59	44	36	50
Bacteremia	12	14	23	14	7	2	5	3	4	0	3	4	2	2	4	9	8	3	7	7	2	4	3	2	4	1	4	0	3	1
Sepsis	3	3	4	2	1	0	0	0	0	0	0	1	0	0	1	5	4	4	4	6	1	1	0	0	1	0	0	0	2	1
Bacteremia Probable SIRS	38	23	32	35	45	13	11	9	10	22	3	5	4	0	10	10	10	5	6	12	6	3	2	3	5	1	1	0	1	2
Probable Septic Shock	12	14	14	5	5	0	1	0	0	0	2	3	2	1	2	17	14	8	15	15	0	1	0	1	0	3	1	0	1	0
Septic Shock	2	1	2	0	2	0	0	0	0	1	1	0	0	0	0	0	3	1	3	1	0	0	0	0	0	0	0	0	0	1

Supplementary Table 3. A state-specific percentage across all folds of the 5-fold cross-validation results for comparison of the different policy strategies. This table corresponds to Fig. 2A.

	Percent of patients with next state					
	With policy antibiotics			Without policy antibiotics		
**State**	better	same	worse	better	same	worse
No SIRS	38% (276/727)	22% (157/727)	40% (294/727)	28% (167/584)	29% (169/584)	43% (248/584)
SIRS	70% (118/169)	10% (15/169)	20% (36/169)	71% (93/130)	6% (9/130)	23% (28/130)
Probable SIRS	44% (436/988)	33% (327/988)	23% (225/988)	29% (284/957)	47% (454/957)	24% (219/957)
Bacteremia	71% (70/99)	14% (14/99)	15% (15/99)	58% (34/58)	25% (15/58)	17% (9/58)
Sepsis	87% (13/15)	0% (0/15)	13% (2/15)	78% (23/29)	11% (3/29)	11% (3/29)
Bacteremia Probable SIRS	67% (173/260)	25% (65/260)	8% (22/260)	64% (43/67)	28% (19/67)	8% (5/67)
Probable Septic Shock	82% (50/61)	1% (1/61)	17% (10/61)	90% (69/76)	3% (2/76)	7% (5/76)
Septic Shock	78% (7/9)	11% (1/9)	11% (1/9)	88% (8/9)	0% (0/9)	12% (1/9)
**TOTAL**	49% (1143/2328)	25% (580/2328)	26% (605/2328)	37% (721/1910)	35% (671/1910)	28% (518/1910)

Supplementary Table 4. Effect of reduced training set (1399 transitions fixed test set). Dependency of CDSS performance on data size based on stratified reduction of the dataset. Outcome is shown for policy-proposed antibiotic combinations and all other combinations for different states. This table corresponds to Fig. 2C.

	**next state with policy antibiotics**																		**next state without policy antibiotics**																	
	**better**						**same**						**worse**						**better**						**same**						**worse**					
**Training set (%)**	Full	90	85	80	75	50	Full	90	85	80	75	50	Full	90	85	80	75	50	Full	90	85	80	75	50	Full	90	85	80	75	50	Full	90	85	80	75	50
**State**																																				
No SIRS	94	129	125	94	94	129	53	65	61	53	53	102	104	102	99	104	104	65	70	35	39	70	70	35	39	27	31	39	39	53	51	53	56	51	51	27
SIRS	69	69	69	12	12	12	8	8	8	1	1	1	13	13	13	1	1	1	10	10	10	67	67	67	1	1	1	8	8	13	1	1	1	13	13	8
Probable SIRS	218	218	218	205	205	241	111	111	111	98	98	135	117	117	117	107	107	149	61	61	61	74	74	38	108	108	108	121	121	34	52	52	52	62	62	70
Bacteremia	19	19	19	26	26	23	5	5	5	7	7	5	4	4	4	5	5	7	15	15	15	8	8	11	5	5	5	3	3	2	3	3	3	2	2	3
Sepsis	3	3	3	3	3	4	0	0	0	0	0	2	1	1	1	1	1	0	3	3	3	3	3	2	1	1	1	1	1	0	1	1	1	1	1	1
Bacteremia Probable SIRS	52	49	49	52	52	52	17	12	12	17	17	8	8	7	7	8	8	17	14	17	17	14	14	14	7	12	12	7	7	0	0	1	1	0	0	7
Probable Septic Shock	22	36	36	36	36	15	1	1	1	1	1	2	5	7	7	7	7	0	22	8	8	8	8	29	1	1	1	1	1	7	4	2	2	2	2	2
Septic Shock	5	5	4	4	4	1	0	0	0	0	0	0	0	0	0	0	0	0	0	0	1	1	1	4	0	0	0	0	0	1	1	1	1	1	1	0
**TOTAL**	482	528	523	432	432	477	195	202	198	177	177	255	252	251	348	233	233	239	195	149	154	245	245	200	162	155	159	180	180	110	113	114	117	132	132	118

Supplementary Table 5. Summary of the reduced training set results. This table corresponds to Fig. 2C.

**Training set**	With policy antibiotics			Without policy antibiotics		
	better	same	worse	better	same	worse
Full	52% **(482/929)**	21% **(195/929)**	27% **(252/929)**	41% **(195/470)**	34% **(162/470)**	25% **(113/470)**
90%	54% **(528/981)**	21% **(202/981)**	25% **(251/981)**	36% **(149/418)**	37% **(155/418)**	27% **(114/418)**
85%	54% **(523/969)**	21% **(198/969)**	25% **(248/969)**	36% **(154/430)**	37% **(159/430)**	27% **(117/430)**
80%	51% **(432/842)**	22% **(177/842)**	27% **(233/842)**	43% **(245/557)**	33% **(180/557)**	24% **(132/557)**
75%	51% **(432/842)**	22% **(177/842)**	27% **(233/842)**	43% **(245/557)**	33% **(180/557)**	24% **(132/557)**
50%	49% **(477/971)**	26% **(255/971)**	25% **(239/971)**	46% **(200/428)**	26% **(110/428)**	28% **(118/428)**

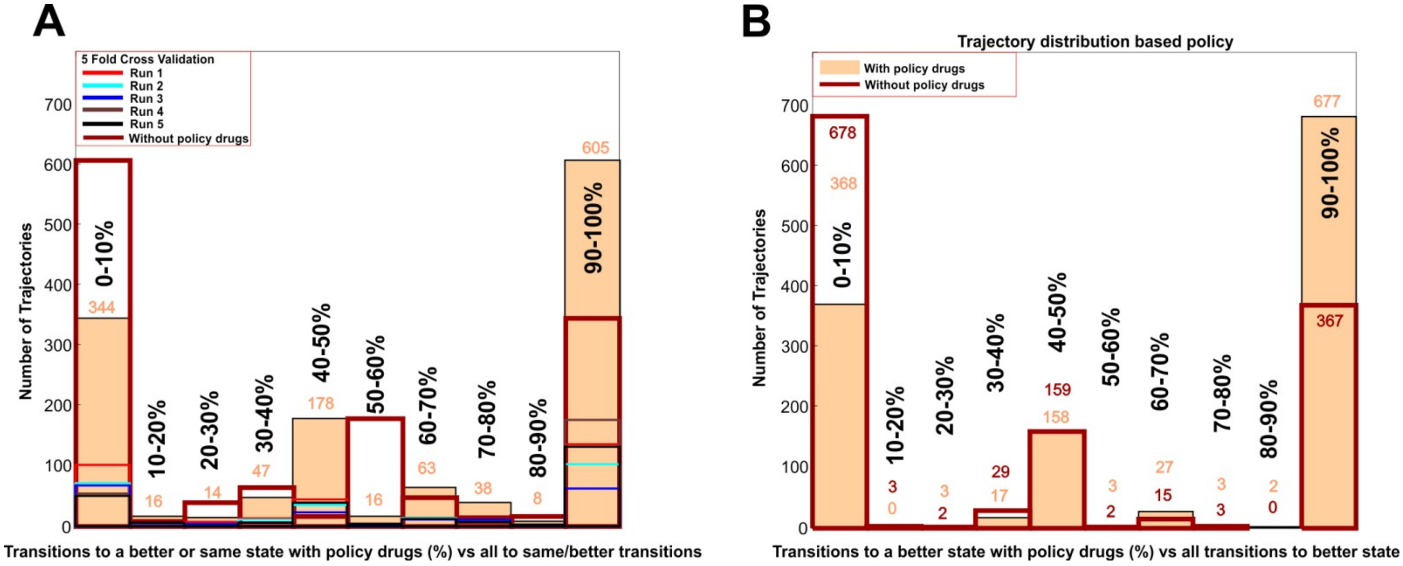

Supplementary Figure 1. Performance and Robustness of the POMDP Clinical Decision Support System for Sepsis. (A) Number of patient trajectories vs. the percentage of their transitions that lead to a better or same state vs all same/better state transitions. Each line represents a different fold across the 5-fold cross-validation. **(B)** Number of patient trajectories vs. the percentage of their transitions that lead to a better state vs all to better state transitions.

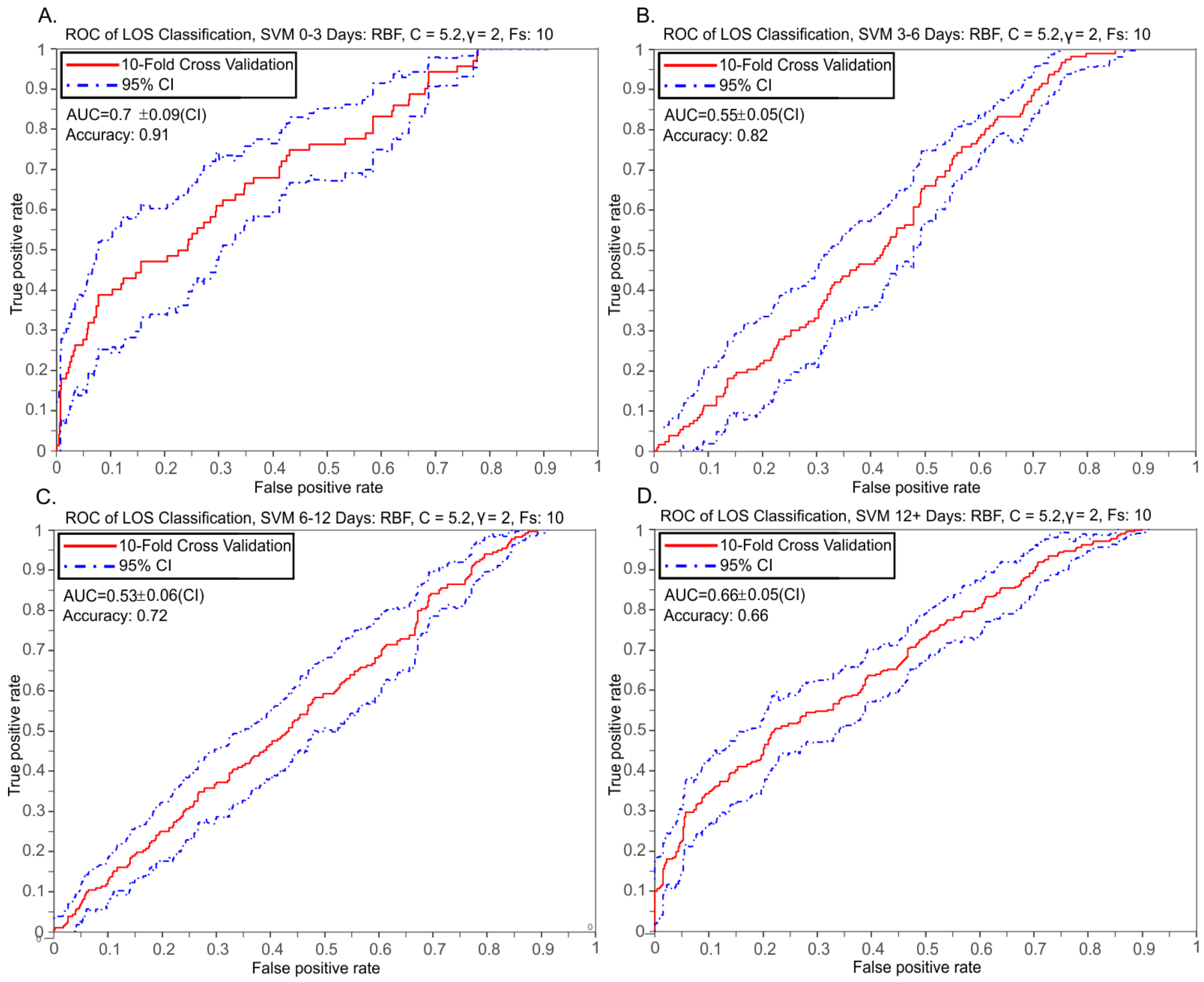

Supplementary Figure 2. Predicting a patient's Length of Stay (LOS) – Multiclass SVM.

ROC curves for a multiclass classifier where 0-3, 3-6, 6-12 and 12+ days groups were used as classes. The one vs all method is used for the training and test group classification in each of the cross validation iterations.

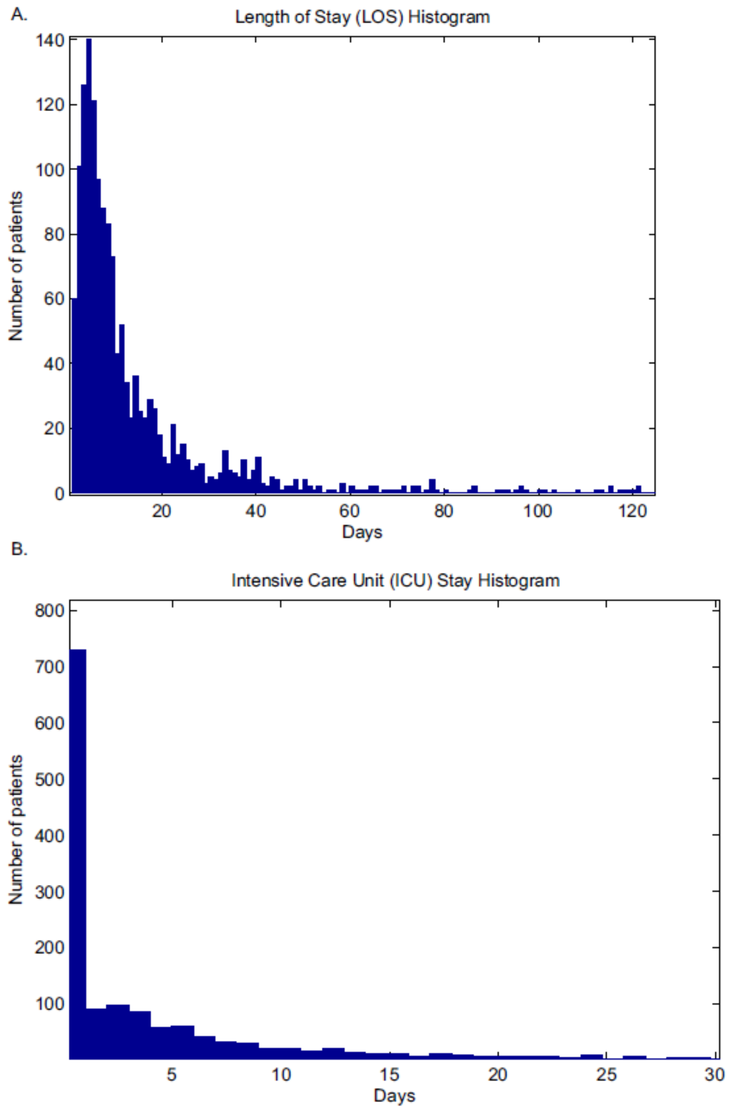

Supplementary Figure 3. Total and ICU length of stay histogram.
